# Radiomics characterization of tissues in an animal brain tumor model imaged using dynamic contrast enhanced (DCE) MRI

**DOI:** 10.1038/s41598-023-37723-8

**Published:** 2023-07-02

**Authors:** Hassan Bagher-Ebadian, Stephen L. Brown, Mohammad M. Ghassemi, Tavarekere N. Nagaraja, Benjamin Movsas, James R. Ewing, Indrin J. Chetty

**Affiliations:** 1 Department of Radiation Oncology, Henry Ford Health, Detroit, MI 48202 USA; 2 Department of Radiology, Michigan State University, East Lansing, MI 48824 USA; 3 Department of Osteopathic Medicine, Michigan State University, East Lansing, MI 48824 USA; 4 Department of Physics, Oakland University, Rochester, MI 48309 USA; 5 Department of Radiation Oncology, Wayne State University, Detroit, MI 48202 USA; 6 Department of Computer Science and Engineering, Michigan State University, East Lansing, MI 48824 USA; 7 Department of Neurosurgery, Henry Ford Health, Detroit, MI 48202 USA; 8 Department of Neurology, Henry Ford Health, Detroit, MI 48202 USA; 9 Department of Neurology, Wayne State University, Detroit, MI 48202 USA

**Keywords:** Cancer models, Computational science, Computational biology and bioinformatics, Data processing, Tumour heterogeneity

## Abstract

Here, we investigate radiomics-based characterization of tumor vascular and microenvironmental properties in an orthotopic rat brain tumor model measured using dynamic-contrast-enhanced (DCE) MRI. Thirty-two immune compromised-RNU rats implanted with human U-251N cancer cells were imaged using DCE-MRI (7Tesla, Dual-Gradient-Echo). The aim was to perform pharmacokinetic analysis using a nested model (NM) selection technique to classify brain regions according to vasculature properties considered as the source of truth. A two-dimensional convolutional-based radiomics analysis was performed on the raw-DCE-MRI of the rat brains to generate dynamic radiomics maps. The raw-DCE-MRI and respective radiomics maps were used to build 28 unsupervised Kohonen self-organizing-maps (K-SOMs). A Silhouette-Coefficient (SC), k-fold Nested-Cross-Validation (k-fold-NCV), and feature engineering analyses were performed on the K-SOMs’ feature spaces to quantify the distinction power of radiomics features compared to raw-DCE-MRI for classification of different Nested Models. Results showed that eight radiomics features outperformed respective raw-DCE-MRI in prediction of the three nested models. The average percent difference in SCs between radiomics features and raw-DCE-MRI was: 29.875% ± 12.922%, p < 0.001. This work establishes an important first step toward spatiotemporal characterization of brain regions using radiomics signatures, which is fundamental toward staging of tumors and evaluation of tumor response to different treatments.

## Introduction

Radiomics analysis can be viewed as an analytical core or a series of algorithmic pipelines that extract structured, meaningful, and mineable information from medical images to reveal subtle characteristic of the data for the prediction, diagnosis, prognosis, and monitoring of different diseases^1–6^. The entire pipeline relies heavily on the hypothesis that medical images contain abundant information and “Images are Data”^5^, which can present an inference of underlying pathobiology of the region of interest^1,5^. Various radiomics analyses and adaptive models (AMs) have been developed to analyze multiparametric MR images of glioblastoma (GBM) to predict outcomes of clinical interest, such as recurrence and survival^7–16^, response to treatment^7,8,13,14,17^, molecular mutation status^18–22^, and subclinical peritumoral infiltration^22–28^. Radiomics has shown considerable success in the prediction of noninvasive biomarkers of outcome^5,29^, quantification and tissue characterization in the field of dynamic contrast enhanced (DCE) MRI^30–41^, and association of imaging biomarkers to biological mechanisms^5,42,43^. However, little progress has been made toward modeling and relating radiomics biomarkers to those fluxes, vasculature physiology, and flows that are known to be significant in steering tumor growth and invasiveness^44–54^. Such an understanding is critically needed for the timely selection of therapies^44,55–58^ in the shifting clinical presentation that is often found in many aggressive tumors. Such a development is a key step toward identifying those radiomics features that can be associated with the physiology and microenvironment of the tumor and its surrounding tissues in human cancer studies.

DCE-MRI has been established as a useful, noninvasive, and non-ionizing method for the quantitative evaluation and characterization of tumor microvasculature and permeability parameters^59–69^. DCE-MRI experiment is performed by applying a fast T1-weighted MR pulse sequence to repeatedly image the volume of interest during the intravenous injection of a contrast agent (CA).

In animal models, DCE-MRI pharmacokinetic (PK) analysis shows promise as a stable descriptor of tumor physiology^59,70^, and of physiological reaction to treatments^61,71–76^. Our group has taken an important step toward the improvement of the image-based estimation of vascular permeability parameters by introducing a nested model selection (NMS) technique^64,77^. The NMS utilizes an extended Patlak graphical method that illuminates PK compartmental analyses of DCE-MRI data from rat and human brains. We have shown^64,77^ that in the PK analysis of the DCE-MRI information of rat and human brains, parsimony is an accepted heuristic^78^. The simplest model with less complexity that fits the data, is considered as an accepted model or the best model to meaningfully explain the variation and characteristics of the data. The parameters of the simplest or parsimonious acceptable model are then taken as the best parameters to summarize the pathophysiological behavior of the underlying tissue^64,77,78^.

**Figure 1:**
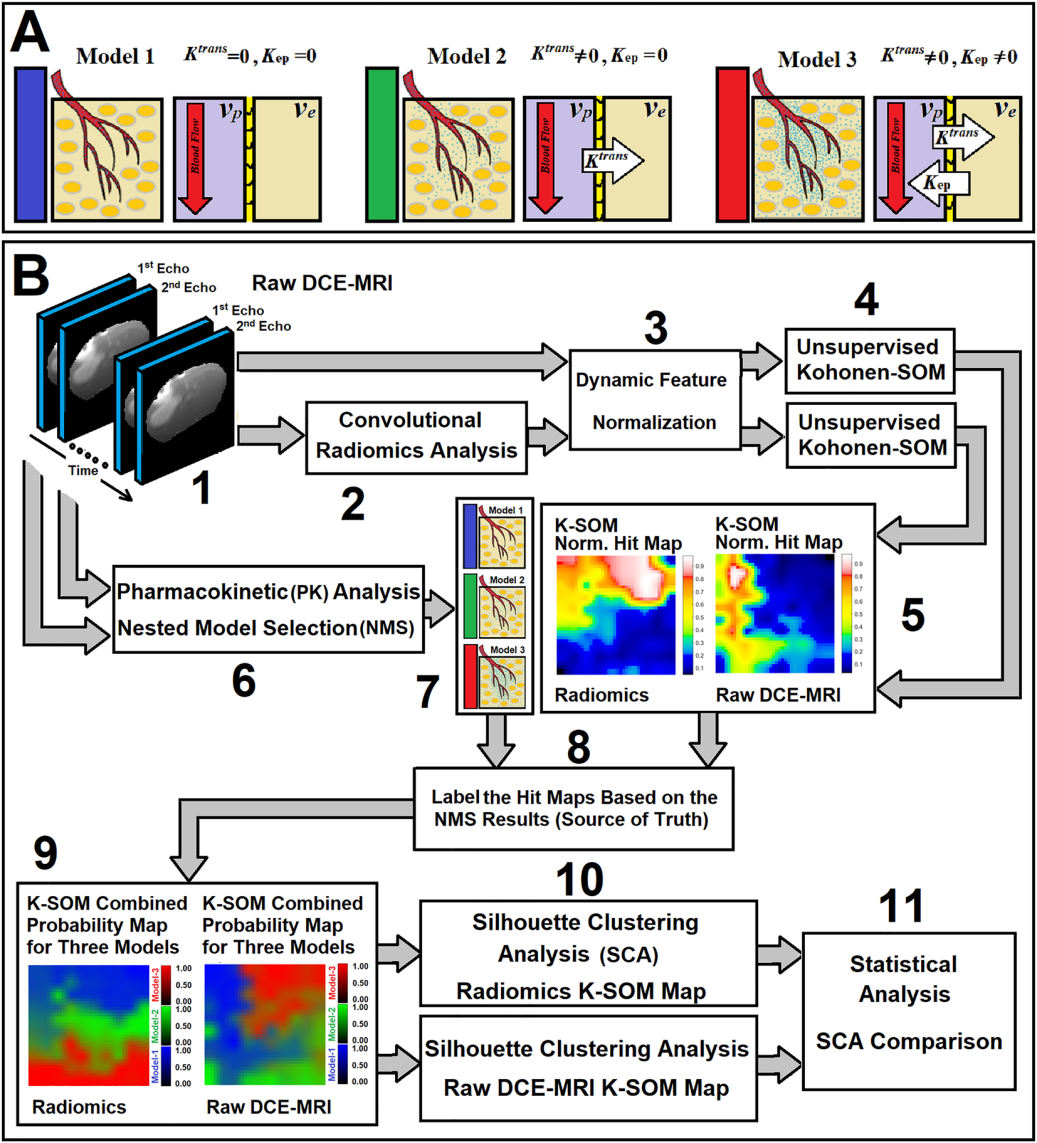
Subfigure (**A**) illustrates three physiologically nested models, their vascular and extra-cellular-extra vascular compartments, and their estimable vascular parameters corresponding to three different observation Eqs. (10–(12). Subfigure (**B**) shows all different stages of the data analysis in this study.

The NMS-based PK analysis^64,77^ physiologically associates the estimable vascular permeability parameters to the relevant information available in the DCE-MRI signal. The NMS-based PK analysis classifies the brain regions into one of the following three different pathophysiologically nested models: Model 1 region: tissues with normal vasculature with no evidence of CA leakage to interstitium space, Model 2 region: tissue vasculature with CA leakage to interstitium space without measurable back-flux to the vasculature compartment, or Model 3 region: tissues vasculature with CA leakage to the interstitium space and measurable back flux to the vasculature compartment (see Fig. 1A).

The NMS concept^64,77^ allows for a selection of an optimal PK model with a less biased estimate of its physiological parameters best fitted to the DCE-MR information, which results in a more accurate and robust characterization of tumor physiology and its microenvironmental parameters.

As a proof of concept, this pilot study analyzes radiomics features from DCE-MR images in an animal model of cerebral tumors to investigate association of radiomics biomarkers with tumor physiology. The goal is to characterize the tumor microenvironment to identify significant image information relevant to pathophysiological properties of brain tissues derived by the NMS-based PK analysis.

## Methods

### Animal population and DCE-MR Imaging

Following previously published methods, and using an institutionally approved protocol, 32 immune-compromised-RNU/RNU rats were stereotactically implanted with 5 × 10^5^ U-251N cancer cells (The U-251N cells^79^ were received as a gift from Dr. Tom Mikkelsen, Department .of Neurosurgery, Henry Ford Health System) in 10 µl of phosphate buffered saline (PBS) that formed a 3–4 mm diameter orthotopic glioma about two weeks later^80^. For each rat, a DCE-MRI study (multi-slice, multi-echo gradient-echo sequence, with three 2.0 mm slices, no gap, matrix:128 × 64, FOV:32 × 32 mm^2^, T_R_/T_E1_/T_E2_ = 24 ms/2 ms/4 ms, flip-angle = 18°, SW = 150 kHz) was performed using a 7 T Varian (Agilent, 20 cm bore system with Bruker console) scanner. Four-hundred slice packages at ~ 1.55 s intervals (total experiment duration: 10 min) were acquired. A bolus of MR contrast-agent (MR CA-Magnevist) was injected (tail-vein) by hand push at acquisition 15. Two T1 mapping (TOMROP, T One by Multiple Read Out Pulses, or Look-Locker, LL) sequences^81,82^ were run to produce voxel-by-voxel maps of tissue T_1_ prior to, and immediately after the Dual Gradient Echo (DGE) DCE-MRI study. All experiments in this study were performed in accordance with relevant guidelines and regulations (IACUC and NMR lab of Henry Ford Health System).

### Nested model selection and observation equations for pharmacokinetic analysis

The Standard Model (SM) is a starting point for evaluating cerebral physiology^64,77,83^ following measurement of contrast tracers using DCE-MRI experiment. Modeling of leakage in the vasculature system in integral form was first generated by Patlak^84,85^ and further modified by Tofts et al.^86,87^. After intravenous administration of CA, the CA concentration in tissue is given by:

1 $${\mathrm{C}}_{t}(t)={K}^{trans}{\int }_{0}^{t}{{\mathrm{e}}^{-{K}_{ep}(\mathrm{t}-\uplambda )}\mathrm{C}}_{p}\left(\lambda \right)d\lambda +{v}_{p}{C}_{p}(t)$$

where: C_p_ and C_t_ are the plasma and tissue concentrations of the CA, K^trans^ is the volumetric forward transfer rate of the indicator into the interstitial space. The k_ep_ in Eq. (1) refers to the transfer rate from the interstitial compartment to the vascular compartment, t and λ denote the experiment time, and v_p_ is the fractional volume of the vascular distribution space, usually thought to be the plasma distribution space. As shown in Fig. 1A, we have shown^64,77^ that three physiologically nested models can be derived from the standard Toft’s model to describe possible physiological conditions of underlying tissue pathology. We have generated a series of stable processing pipelines accordingly, for producing vascular parametric maps based on the NMS^64,77^. We assume that CA concentration, [Gd], is proportional to the change in the longitudinal relaxation rate (R_1_) after CA administration: [Gd] = CA(t) ~ ΔR_1_(t), where R_1_ = 1/T_1_ (T_1_ is the longitudinal relaxation time). This assumption is supported in most DCE-MRI studies because they are carried out under the conditions of rapid repetition time and Ernst tip-angle adjusted for a mean decrease in R_1_. Under these conditions, studies in multicompartmental systems demonstrate little effect of exchange mechanisms^88,89^.

### Calculation of CA concentration (∆R_1_) from the dual gradient echo pulse sequence

This section describes the calculation of [ΔR_1_] as it is estimated from the data of the dual gradient echo (DGE) that is sandwiched between two TOMROP or Look-Locker pulse sequences. DGE imaging allows computation of *T*_*1*_ and *T*_2_* as approximately pure and independent components. This is essential for estimation of CA concentration based on *ΔR*_*1*_ and *ΔR*_2_*. Below, we will describe the method we have developed for calculating the *ΔR*_*1*_ signal (~ CA concentration) from the DGE signals. The following equations describe the measured T_1_-weighted intensities of the first and second echo signals and their relationship with the longitudinal and transverse relaxation times (*T*_*1*_*, T*_2_^*^), repetition time (*T*_*R*_), echo time (*T*_*E*_), the flip angle (*θ*), and the equilibrium longitudinal magnetization (*M*_*0*_). *F* and S in these equations denote the signal from the 1st and 2nd echoes, respectively:

2 $$F=\frac{{M}_{0}\mathrm{sin}\theta \left(1-{e}^{\frac{-TR}{T1}}\right){e}^{\frac{-TE1}{{T}_{2}^{*}}}}{1-{\mathrm{cos}\theta e}^{\frac{-TR}{T1}}}$$

3 $$S=\frac{{M}_{0}\mathrm{sin}\theta \left(1-{e}^{\frac{-TR}{T1}}\right){e}^{\frac{-\mu TE2}{{T}_{2}^{*}}}}{1-{\mathrm{cos}\theta e}^{\frac{-TR}{T1}}}$$

4 $$\mu =\frac{TE2}{TE1}$$

For each voxel, there are two main phases for the signal: the pre-injection phase and the post injection phase. To set a baseline for the signal intensity of each voxel prior to the injection of the CA, the voxel intensities for the ${S}_{t}$ and ${F}_{t}$ signals are averaged over a few time points prior to injection:

5 $${{F}^{pre}=\frac{1}{(n-m+1)}\sum_{t=m}^{n}{F}_{t}, F}^{sat}=\frac{1}{(p-o+1)}\sum_{t=o}^{p}{F}_{t}$$

6 $${S}^{pre}=\frac{1}{(n-m+1)}\sum_{t=m}^{n}{S}_{t}, { S}^{sat}=\frac{1}{(p-o+1)}\sum_{t=o}^{p}{S}_{t}$$

In Eqs. (5) and (6), t denotes the experiment time point and ${\mathrm{F}}^{\mathrm{pre}}$, and ${\mathrm{S}}^{\mathrm{pre}}$ are the mean of the signal intensities associated with first and second echoes between time points m and n, where m and n are smaller than the injection time. In these equations, ${\mathrm{F}}^{\mathrm{Sat}}$, and ${\mathrm{S}}^{\mathrm{Sat}}$ are the mean of the signal intensities associated with first and second echoes (saturated part of the signals) between time points o and p, where p is the last time point of the signals (post injection), and p minus o is equal to n minus m (p–o = n–m).

In these equations, *t* denotes the experiment time point and ${F}^{pre}$ and ${S}^{pre}$ are the mean of the signal intensities associated with first and second echoes between time points *m* and *n,* where *m* and *n* are smaller than the injection time. Also, we define ${E}^{pre}$ and ${E}^{post}$ as:

7 $${{E}^{pre}=e}^{\frac{-TR}{T1(pre)}},{{E}^{post}=e}^{\frac{-TR}{T1(post)}}$$

which are *T*_*1*_ associated parameters, estimated from the *T*_*1*_ mapping pulse sequences that are run before and after the DCE-MRI study. Using the above definitions, the following equations for the preprocessing of the DGE signals for DCE imaging are derived:

8 $${\theta }_{\mathit{Est}}= {\mathrm{cos}}^{-1}\left\{\frac{1-\left[\frac{{\left( \sum_{t=m}^{n}{S}_{t}^{pre} \right)}^{ \frac{1}{\mu -1}}}{{\left( \sum_{t=m}^{n}{F}_{t}^{pre} \right)}^{ \frac{\mu }{\mu -1}}}\right]\left[\frac{{\left( \sum_{t=m}^{n}{F}_{t}^{sat} \right)}^{ \frac{\mu }{\mu -1}}}{{\left( \sum_{t=m}^{n}{S}_{t}^{sat} \right)}^{ \frac{1}{\mu -1}}}\right]}{{E}^{post}-{E}^{pre}\left[\frac{{\left( \sum_{t=m}^{n}{S}_{t}^{pre} \right)}^{ \frac{1}{\mu -1}}}{{\left( \sum_{t=m}^{n}{F}_{t}^{pre} \right)}^{ \frac{\mu }{\mu -1}}}\right]\left[\frac{{\left( \sum_{t=m}^{n}{F}_{t}^{sat} \right)}^{ \frac{\mu }{\mu -1}}}{{\left( \sum_{t=m}^{n}{S}_{t}^{sat} \right)}^{ \frac{1}{\mu -1}}}\right] }\right\}$$

9 $${\Delta R}_{1}\left(t\right)= \frac{-1 }{TR}\mathrm{ln}\left\{\frac{1}{\mathrm{cos}{\theta }_{Est}{E}^{pre}}+\left(n-m+1\right)\left[1- \frac{1}{\mathrm{cos}{\theta }_{Est}{E}^{pre}}\right]\left\{\frac{{\left[ \sum_{t=m}^{n}{S}_{t}^{pre} \right]}^{ \frac{1}{\mu -1}}}{{\left[ \sum_{t=m}^{n}{F}_{t}^{pre} \right]}^{ \frac{\mu }{\mu -1}}}\right\}\left\{\frac{{\left[{ F}_{t} \right]}^{ \frac{\mu }{\mu -1}}}{{\left[{ S}_{t} \right]}^{ \frac{1}{\mu -1}}}\right\}\right\}$$

As seen in these equations, for each voxel, the pure component ${\Delta R}_{1}\left(t\right)$, along with its actual tip angle are estimated. Of note, one of the interesting observations about this equation is that the estimated ${\Delta R}_{1}\left(t\right)$ is fully independent from the value of ${E}^{post}$. In this study, the voxel-wise profiles of the CA concentration map (${\Delta R}_{1}\left(t\right)$) were directly estimated from these equations and the pre and post *T*_*1*_ associated parameters (${E}^{pre}$, and ${E}^{post}$) during the DCE-MRI experiment.

In this study, given Eq. (1), the time trace of change in the longitudinal relaxation time (ΔR_1_) in all the voxels of the animal’s brain for 32 DCE-MRI studies were calculated^77^. Post-processing and pharmacokinetic compartmental analyses of DCE-MRI data were carried out following published methods^64,77^, initially using a nested model selection (NMS) paradigm based on Patlak and extended Patlak graphical method^84,85^. As shown in Fig. 1A, the NMS technique^64,77^ was used to generate maps of brain regions labeled with the number of parameters used to describe the data: (a) Model 1 region: normal vasculature with no leakage, the only parameter estimated is plasma volume, v_p_; (b) Model 2 region: tumor tissues with CA leakage without measurable back-flux to the vasculature, in which case, v_p_ and, K^trans^ can be estimated; or (c) Model 3 region: tumor vessels with CA leakage and measurable back-flux and, thus, v_p_, K^trans^, and k_ep_, or extracellular extra-vascular volume, v_e_ (ratio of K^trans^ and K_ep_) can be estimated. Three observation equations [see Eqs. (10)–(12)] corresponding to three physiologically nested models were constructed from Eq. (1) as follows:

10 $${\mathrm{C}}_{tissue}(\mathrm{t})=\frac{1}{1-{H}_{ct}}[{{v}_{p}\mathrm{C}}_{AIF}\left(\mathrm{t}\right)]$$

11 $${\mathrm{C}}_{tissue}(\mathrm{t})=\frac{1}{1-{H}_{ct}}[{{v}_{p}\mathrm{C}}_{AIF}\left(\mathrm{t}\right)+{K}^{trans}{\int }_{0}^{t}{\mathrm{C}}_{AIF}\left(\lambda \right)d\lambda ]$$

12 $${\mathrm{C}}_{tissue}(\mathrm{t})=\frac{1}{1-{H}_{ct}}[{{v}_{p}\mathrm{C}}_{AIF}\left(\mathrm{t}\right)+{K}^{trans}{\int }_{0}^{t}{\mathrm{C}}_{AIF}\left(\lambda \right){e}^{-{K}_{ep}(t-\lambda )}d\lambda ]$$

where *C*_*AIF*_* (t) and C*_*tissue*_* (t)* refer to the contrast agent (CA) concentration measured from plasma (or arterial input function, AIF) and tissue of interest in the brain, respectively. The *Hct* in these equations refers to the hematocrit ratio of animal’s blood.

For each observation equation, the voxel by voxel time trace of ${\Delta \mathrm{R}}_{1}\left(\mathrm{t}\right)$ in rat brain was used to estimate its PK parameters as well as constructing an AIF for the brain^90^, based on a group averaged radiological trace of CA concentration in arterial blood^90^. At image 15 (corresponds to ~ 23 s after the start of the DCE experiment) of the dual GE sequence, a bolus injection of the CA (Magnevist; Bayer HealthCare LLC, Wayne, NJ, 0.25 mmol/kg at undiluted concentration, no flush) was performed. The group averaged radiological trace was normalized to the time trace of CA concentration in the animal’s normal caudate putamen^90^, with the assumption that plasma volume fraction in caudate putamen is 1%. Then, the normalized radiological AIF was used as C_p_ (CA concentration measured from plasma) or $\frac{{\mathrm{C}}_{\mathrm{AIF}}}{1-\mathrm{Hct}}$ in Eqs. (10)–(12). A Simplex optimization/algorithm^91^ was used to estimate PK parameters for each model. Our experience^59,60,63,64,77,89,92–99^ with the Simplex optimization is that it is time-intensive but very reliable in converging to a best-fit value.

### DCE-MRI data pre-processing, data quantization, and radiomics analysis

First and second echoes of DCE-MRI information for all animals were normalized as follows: for each voxel of the rat brain, the last 10 timepoints of its 1st echo profile, corresponding to the stable portion (plateau) of the signal, were averaged and used as the normalization factor to normalize the 1st and 2nd echo profiles. The normalized profiles (1st and 2nd echoes) were quantized and uniformly resampled into 128 intensity levels using a Fixed Bin Number (FBN^100,101^) according to the recommendations provided by the image biomarker standardization initiative (IBSI)^100–102^. A 2D-convolutional (symmetrical window, 5 × 5) radiomics analysis was performed on the normalized and resampled DCE-MRI data (for all 400 time points of the three slices of the 1st and 2nd echo images) of all 32 animals. The radiomics analysis was preformed using our in-house-developed ROdiomiX software system^103,104^, which consists of a series of computational cores designed and validated on the recommendations provided by the IBSI^100,101^. A series of IBSI-validated convolutional radiomics feature maps (133 feature maps, window size = 5X5, Chebyshev distance = 1.0, with 8-connected neighborhoods) from eight different feature categories (see Table 1) were computed for each time point of the three slices using the following three IBSI recommended aggregation techniques (see column 5 of Table 1): (1) DHQ4: features were calculated over the volume of interest. (2) BTW3: features were computed from each 2D directional matrix and averaged over 2D directions. (3) 8QNN: features were directly computed from 2D matrices^100,101^.

**Table 1:** This table shows eight different IBSI-validated radiomics feature categories, number of radiomics features in each category, and their IBSI aggregation method.

No.	Feature category	Abbressssviation	No of features	IBSI aggregation
1	Intensity histogram	IH	21	2D-averaged (DHQ4)
2	Intensity based statistical	IBS	17	2D-averaged (DHQ4)
3	Gray level co-occurrence matrix	GLCM	25	2D-averaged (BTW3)
4	Gray level run length matrix	GLRLM	16	2D-averaged (BTW3)
5	Gray level size zone matrix	GLSZM	16	2D-averaged (8QNN)
6	Gray level distance zone matrix	GLDZM	16	2D-averaged (8QNN)
7	Neighborhood gray tone difference matrix	NGTDM	5	2D-averaged (8QNN)
8	Neighboring gray level dependence matrix	NGLDM	17	2D-averaged (8QNN)

As shown in this table, the total number of radiomics features for all categories was 133.

### Feature engineering, Kohonen self-organizing map, and clustering analysis

To suppress the variations of the MR signal information due to the instability of the MR scanner’s gain at the beginning of the DCE-MR imaging, the first 10 timepoints of all dynamic maps (corresponding to the pre injection part of the signal for all animals which contains no contrast enhanced information: unstable part of the signal) were excluded from the analysis. Thus, for each voxel of the rat brain, 134 information profiles corresponding to 133 dynamic radiomics information modalities (time-trace of radiomics information) and one raw DE-MRI information for each echo with a length of 390 timepoints were selected and used as dynamic feature sets for feature engineering analysis. The 390 timepoints corresponded to the acquired information from 15.5 to 620 s of the DCE-MRI experiment.

For each information modality (from 268 dynamic maps for both echoes), the normalized contrast-enhancement-ratio (Norm-CER)^105^ between the tumor volume and normal area (for all feature maps extracted from the 1st echo and corresponded to Model 1 and Models 2 & 3, respectively, see Section “Nested model selection and observation equations for pharmacokinetic analysis”) of the rat brain was calculated. The Norm-CER value was averaged over 32 studies and used to exclude the radiomics information modalities with low normalized contrast enhancement ratio (NCER < 1.2) that were not DCE informative compared to the raw DCE MR information^105^.

For each information modality, extracted from each echo, 143,057 information profiles were selected from all the rat brain voxels and used to build 28 different unsupervised Kohonen self-organizing maps (K-SOMs) corresponding to 26 radiomics feature maps (13 dynamic feature maps extracted from 1st echo and 13 dynamic feature maps extracted from 2nd echo) and two raw DCE-MRI maps (1st and 2nd echoes). The processing details are shown in Fig. 1B.

A Kohonen self-organizing map (K-SOM)^106,107^ or self-organizing feature map is an unsupervised machine learning technique used to produce a low-dimensional representation of higher dimensional data set with complex structures while preserving the topological structure of the data. During the K-SOM analysis, a competitive learning algorithm^108–110^ along with Best Matching Unit (BMU) strategy were employed to identify the winner nodes/neurons for a 10 × 10 topology. The cover steps for initial covering of the input space for ordering the phase steps of the K-SOM was set to 100. The K-SOM’s architecture was hexagonal with an initial neighborhood size of three, and the maximum epoch was set to 200 epochs for batch training mode.

A BMU-based hit map was generated for each information modality (see Fig. 1B). The model choice labels (corresponding to three different models) estimated from PK-NMS analyses (see Section “Nested model selection and observation equations for pharmacokinetic analysis” and Fig. 1B) for all profiles of different information modalities were used as the source of truth to calculate the tagged BMUs on each K-SOM topology. For each information modality (28 maps, corresponding to 1st and 2nd echoes and their 13 respective radiomics maps), the hit maps and their corresponding labels were used to estimate three K-SOM probability and iso-probability maps for three different model choices. To investigate the information content of each radiomics features to perform NMS on DCE-MRI data, and to reveal non-linearity in the clusters and their associations^106,107^with the physiological state (identified by the NMS analysis, see Section “Nested model selection and observation equations for pharmacokinetic analysis”), an unsupervised Silhouette clustering analysis^111,112^ was performed on the K-SOM feature space.

Silhouette coefficients near + 1 indicate that the samples are far away from the neighboring clusters. A value of 0 indicates that the sample is on or very close to the decision boundary between the two neighboring clusters and negative values indicate that those samples might have been assigned to a wrong cluster. A variable threshold (from 0.01 to one with interval of 0.01, corresponds to 100 probability states) was applied on the K-SOM feature space of each information modality and a Silhouette analysis was performed for each state of probability. Levene’s test^113^ was applied on the mean values of the Silhouette coefficients (SCs) of the K-SOM space for different clusters to test the homoscedasticity (homogeneity of variance) of various SCs on the K-SOM space (1st and 2nd echoes, compared to their corresponding radiomics information). According to the Levene’s test^113^ results (variance homogeneity condition), either an ANOVA or Welch’s test^114^ was used as the null hypothesis test at the 0.95 significance level (see the final step of Fig. 1B, step 11). The Holm–Bonferroni method^115^ was used for circumventing the problem of multiple comparisons for the computed p-values.

The percent difference between the average values of the SCs (DSC% = 100 × [SC_Radiomics_/SC_raw-DCE_ − 1], where SC_Radiomics_ and SC_raw-DCE_ refer to average values of SCs for dominant clusters generated by the radiomics and raw-DCE-MRI information, respectively) measured from radiomics and raw DCE-MRI information was calculated. The DSC% value was used as the distinction power of the radiomics feature compared to the raw DCE-MRI information for classification of the three physiologically nested-models. Different steps of the analysis are fully described in Fig. 1B.

In addition to the Silhouette clustering analysis (SCA), the association of the two raw DCE-MRI information and their respective radiomics information with the three physiologically nested models were evaluated using a k-fold Nested Cross Validation (NCV) technique^116^. We performed a voxel-wise k-fold NCV (k = 10) analysis for the two raw DCE-MRI data (1st and 2nd echoes) and their 13 respective radiomics features extracted from all the rat brain voxels. The full dataset was randomly permuted (using Random Permutation Sampling, RPS^117^) and split into 10 non-overlapping folds. Two independent loops were defined as outer and inner loops. In the outer loop, for each trial, the data was split into two folds (training + validation fold, and a test fold), and for the inner loop, only the validation + training fold was used to construct a series of Kohonen self-organizing maps (K-SOMs). Thus, for each iteration, 28 different unsupervised K-SOMs corresponding to 26 radiomics feature maps (13 dynamic feature maps extracted from 1st echo and 13 dynamic feature maps extracted from 2nd echo) and two raw DCE-MRI maps (1st and 2nd echoes) were constructed in the inner loop using the training cohorts. The trained KSOM constructed in each fold of the inner loop was used as the classifier of the test cohorts of its respective fold in the outer loop. This process was repeated 10 times (k = 10) and at each repetition, an independent test set was withheld for the estimation of the performance of the constructed KSOMs of the inner loop for the three different nested models. Two hundred eighty K-SOMs were constructed (corresponding to 28 information modalities times 10 folds). Since the number of the samples for the three nested models were highly imbalanced, an adjusted AUC, Balanced Accuracy, and F1 score were used as the evaluation metrics to quantify the performance of different K-SOMs for multi-class classification of the three nested models. Similar to the Silhouette clustering analysis, for the ROC analysis of the k-fold NCV, the KSOM’s hit maps were constructed at different specificity levels (or probability thresholds, varying from 0.01 to 1.00, corresponding to 100 probability intervals) based on the NMS concept. A non-parametric Receiver-Operating-Characteristic (ROC) method^118,119^ was used to calculate the adjusted value of the Area-Under ROC (AUC) based on different class labels (multi-class classification). Finally, the micro averaging technique^120^ was recruited to calculate the average values of all the matrices (AUC, BA, and F1 Score) and their respective confidence intervals over all the three models and the NCV’s 10-folds.

### Animal study

This study was approved at the Institutional Animal Care and Use Committee (IACUC) board of Henry Ford Health System and conducted with an approved IACUC # 1509. The animal study of this work was performed and reported in compliance with the ARRIVE guidelines.

## Results

Table 1 shows the eight different IBSI-validated radiomics feature categories, number of radiomics features in each category, and the IBSI aggregation method used for their radiomics feature computation. Fig. 1A shows three different physiologically nested conditions associated with normal tissue and leaky tumor, their respective physiological compartments, and estimable vascular parameters, respectively. Fig. 1B shows the workflow of the data analysis in this study.

**Figure 2:**
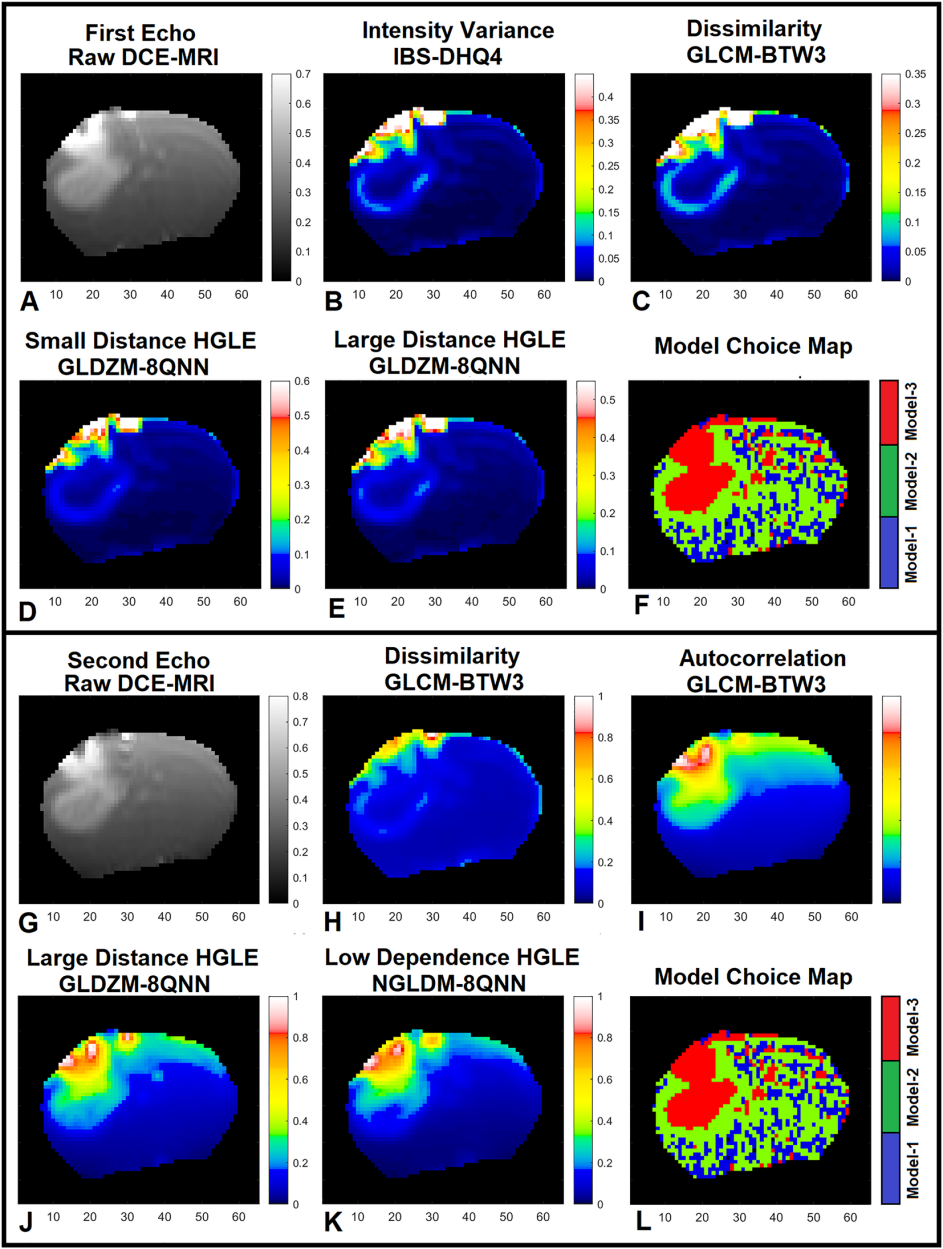
Subfigures (**A**–**F**) demonstrate the 1st echo image of the raw DCE-MRI, its four different significant radiomics maps, and the model choice map estimated by the PK-NMS analysis for a slice of rat brain. Subfigures (**G**–**L**) demonstrate the 2nd echo map of raw DCE-MRI, its four different significant radiomics maps, and conventional model choice map (estimated by the PK-NMS) for the same slice of rat brain at timepoint 100 (~ 2 min).

Figure 2A–F demonstrate the 1st echo image of the raw DCE-MRI, the related 4 different significant radiomics maps, and the model choice map estimated by the PK-NMS analysis (Section“Nested model selection and observation equations for pharmacokinetic analysis”) for a slice of rat brain. Figure 2A–F demonstrate the 1st echo map of raw DCE-MRI, significant radiomics maps, and conventional model choice map (estimated by the PK-NMS, Section “Nested model selection and observation equations for pharmacokinetic analysis”) for a slice of rat brain at timepoint 100 (around 2 min after DCE-MRI experiment). As shown in this figure, compared to the 1st echo information, the following radiomics features significantly distinguished the three model choice regions after administration of CA concentration: (1) Intensity Variance feature (from IBS feature category), (2) Dissimilarity feature (from GLCM feature category), (3) Small Distance High Grey Level Emphasis feature (from GLDZM feature category), and (4) Large Distance High Grey Level Emphasis feature (from GLDZM feature category) with DSC% = 32% (p < 0.001), 49% (p < 0.001), 12% (p < 0.046), and 13% (p < 0.037), respectively. Figure 2G-L demonstrate the 2nd echo map of raw DCE-MRI, the related different significant radiomics maps, and conventional model choice map (estimated by the PK-NMS, Section “Nested model selection and observation equations for pharmacokinetic analysis”) for the same slice of rat brain at timepoint 100 (~ 2 min). As shown in this figure and Table 2, compared to the 2nd echo information, the following radiomics features significantly distinguished the three model choice regions after administration of CA concentration: (1) Dissimilarity feature (from GLCM feature category), (2) Autocorrelation feature (from GLCM feature category), (3) Large Distance High Grey Level Emphasis feature (from GLDZM feature category), and (4) Low Dependence High Grey Level Emphasis feature (from NGLDM feature category) with DSC% = 42% (p < 0.001), 23% (p < 0.001), 32% (p < 0.001), and 28% (p < 0.001), respectively.

**Table 2:** This table shows the radiomics feature category, feature name, DSC%, and p-value of the significant radiomics features extracted from the two raw DCE-MRI information (1st and 2nd echo images).

Significant radiomics features (1st echo)	DSC%	p-value	Significant radiomics features (2nd echo)	DSC%	p-value
Feature category: name			Feature category: name		
IBS: intensity variance	32	< 0.001	GLCM: autocorrelation	23	< 0.001
GLCM: dissimilarity	49	< 0.001	GLCM: dissimilarity	42	< 0.001
GLDZM: small distance high grey level emphasis	12	< 0.046	GLDZM: large distance-high grey level emphasis	32	< 0.001
GLDZM: large distance high grey level emphasis	13	< 0.037	NGLDM: low dependence-high grey level emphasis	28	< 0.001

Among 133 dynamic radiomics feature maps, only 13 dynamic radiomics feature maps met the normalized contrast enhancement ratio criteria^105^ (NCER < 1.2, see Section “Feature engineering, kohonen self-organizing map, and clustering analysis”) and were included and used in the study.

**Figure 3:**
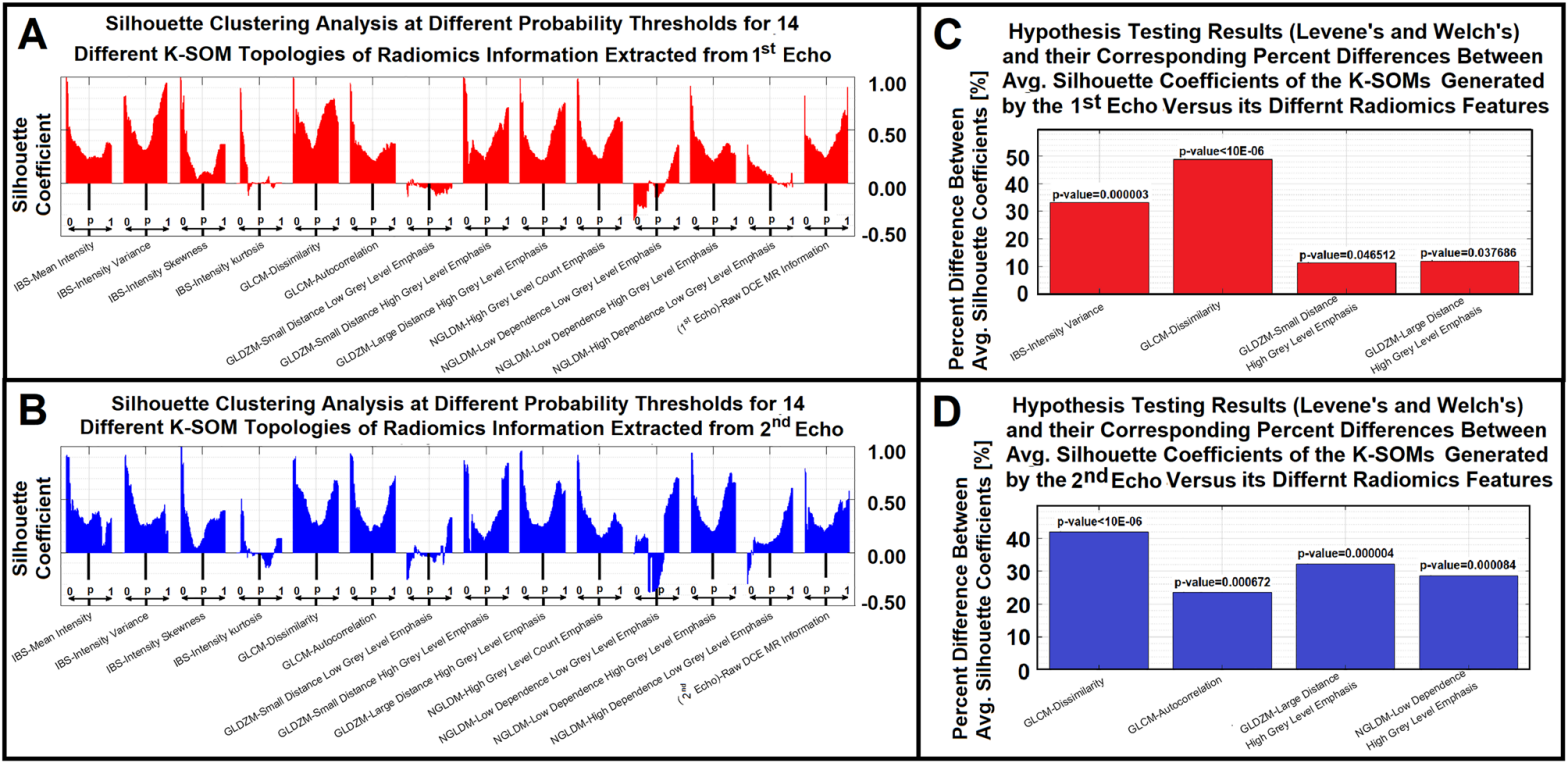
Subfigures (**A**) and (**B**) demonstrate Silhouette clustering analysis of the K-SOM topologies for the 1st and 2nd echoes of DCE-MRI and their respective 13 different radiomics features at different probability thresholds. Subfigure 3C demonstrates the percent difference between the average Silhouette coefficient of the 1st echo K-SOM’s topology and its four radiomics K-SOMs’ topologies that were rejected by the Levene’s and Welch’s hypotheses testing as the significant features. Subfigure 3D demonstrates the percent difference between the average Silhouette coefficient of the 2nd echo K-SOM’s topology and its four radiomics K-SOMs’ topologies that were rejected by the Levene’s and Welch’s hypotheses testing as the significant features.

**Figure 4:**
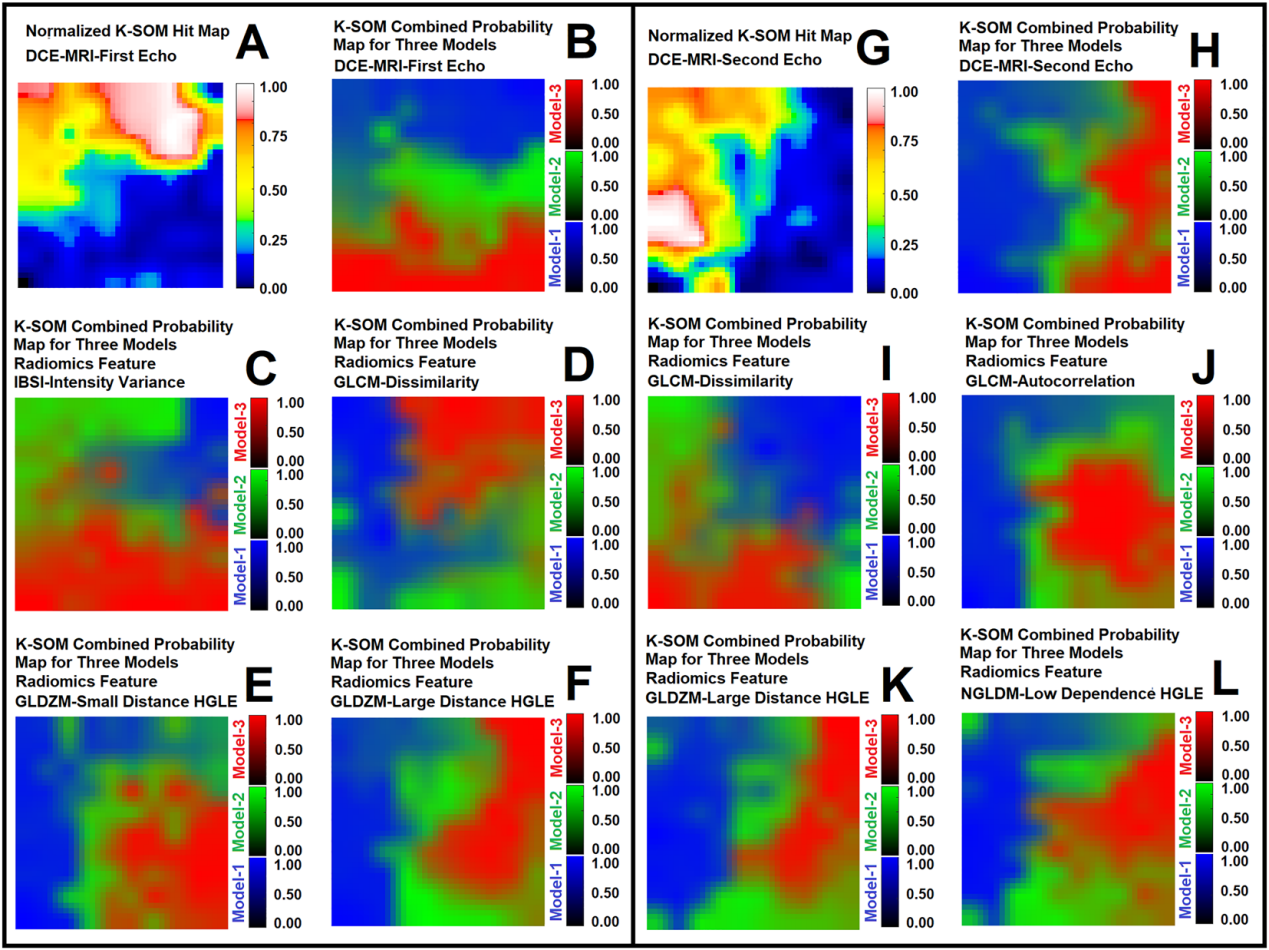
Subfigures (**A**–**F**) demonstrate the normalized K-SOM hit map (K-SOM unsupervised clustering of 143,057 information profiles, Section “Feature engineering, kohonen self-organizing map, and clustering analysis”) for the 1st echo DCE-MR information and the fused probability maps (labeled from conventional PK analysis for the three nested models, Section “Nested model selection and observation equations for pharmacokinetic analysis”) of K-SOM for the 1st echo and its four corresponding significant radiomics feature maps. Subfigures (**G**–**L**) demonstrate the normalized K-SOM hit map (K-SOM unsupervised clustering of 143,057 information profiles, Section “Feature engineering, kohonen self-organizing map, and clustering analysis”) for the 2nd echo DCE-MR information and the fused probability maps (labeled from conventional PK analysis for the three nested models, Section “Nested model selection and observation equations for pharmacokinetic analysis”) of K-SOM for the 2nd echo and its four corresponding significant radiomics feature maps. The blue, green, and red zones on the K-SOM topology maps (**B**–**F** and **H**–**L**) correspond to the models 1, 2, and 3 estimated from NMS-PK analysis, respectively. All the maps are up sampled by four for a better visualization.

Figure 3A and B demonstrate Silhouette clustering analysis of the K-SOM topologies for the 1st and 2nd echoes of DCE-MRI and their respective radiomics features (met the NCER criteria, NCER < 1.2) at different probability thresholds (100 thresholds: varied from 0.01 to 1 with probability interval of 0.01). Subfigure 3C demonstrates the percent difference between the average Silhouette coefficient of the 1st echo K-SOM’s topology and the related radiomics K-SOM topologies that were rejected by the Levene’s and Welch’s tests as the significant features. Figure 3D demonstrates the percent difference between the average Silhouette coefficient of the 2nd echo K-SOM’s topology and related radiomics K-SOMs’ topologies that were rejected by the Levene’s and Welch’s tests as the significant features. Table 3 demonstrates the overall variation of the SCs for each information modality, the mean and standard deviations of the SCs for all information modalities (the two raw DCE-MRI information, 1st and 2nd echoes, and their 13 respective radiomics features) were calculated and reported in this table. Figure 4A–F demonstrate the normalized K-SOM hit map (K-SOM unsupervised clustering of 143,057 information profiles, Section “Feature engineering, kohonen self-organizing map, and clustering analysis”) for the 1st echo DCE-MR information and the fused probability maps (labeled from conventional PK analysis for the three nested models, Section “Nested model selection and observation equations for pharmacokinetic analysis”) of K-SOM for the 1st echo and corresponding significant radiomics feature maps. Figure 4G–L demonstrate the normalized K-SOM hit map (K-SOM unsupervised clustering of 143,057 information profiles, Section “Feature engineering, kohonen self-organizing map, and clustering analysis”) for the 2nd echo DCE-MR information and the fused probability maps (labeled from conventional PK analysis for the three nested models, Section “Nested model selection and observation equations for pharmacokinetic analysis”) of K-SOM for the 2nd echo and corresponding significant radiomics feature maps.

**Table 3:** This table shows the mean and standard deviation values of the Silhouette coefficients (SCs) for the two raw DCE-MR information and their 13 respective radiomics features

Feature category—feature name	1st Echo—SCs (mean ± std)	2nd Echo SCs (mean ± std)
Raw DCE MR information	0.392 ± 0.162	0.332 ± 0.131
IBS-mean intensity	0.336 ± 0.146	0.358 ± 0.183
IBS-intensity variance	0.522 ± 0.226	0.368 ± 0.187
IBS-intensity skewness	0.235 ± 0.215	0.275 ± 0.202
IBS-intensity kurtosis	0.067 ± 0.195	0.022 ± 0.123
GLCM-dissimilarity	0.583 ± 0.154	0.472 ± 0.177
GLCM-autocorrelation	0.342 ± 0.141	0.411 ± 0.202
GLDZM-small distance low grey level emphasis	− 0.050 ± 0.037	− 0.000 ± 0.128
GLDZM-small distance high grey level emphasis	0.435 ± 0.201	0.377 ± 0.261
GLDZM-large distance high grey level emphasis	0.438 ± 0.201	0.439 ± 0.191
NGLDM-high grey level count emphasis	0.429 ± 0.175	0.313 ± 0.171
NGLDM-low dependence low grey level emphasis	− 0.037 ± 0.184	0.131 ± 0.352
NGLDM-low dependence high grey level emphasis	0.341 ± 0.132	0.427 ± 0.209
NGLDM-high dependence low grey level emphasis	0.079 ± 0.087	0.158 ± 0.186

Figure 4A and G demonstrate the K-SOM’s normalized hit map for all the voxels of the 32 rat brains measured from the raw-DCE-MR information of the 1st and 2nd echoes, respectively. The K-SOM hit maps represent the distribution of the data structures and their similarities on the feature space. Each point on the map is associated with unique properties of a signal (time trace of information), and its value corresponds to the normalized number of the winning BMU (best matching unit) for that specific signal characteristics. On the feature space, the adjacent points represent similar characteristics while distant points show the highest dissimilarities.

Figure 4B–F and H–L illustrate the probabilities of different models for different information modalities calculated from their respective K-SOM hit maps and labeled according to the associated nested model selection information (source of truth). Figure 4B–F and H–L clearly demonstrate how different time traces of information modalities (raw-DCE MRI and their four respective significant radiomics features) correspond to different pathophysiological states of tissue that are grouped together based on their structural similarities at different timepoints. The blue, green, and red zones on the fused K-SOM’s topology maps (Figure 4B–F,H–L) correspond to the models 1, 2, and 3, estimated from the NMS-PK analysis, respectively.

The blue, green, and red zones on the K-SOM topology maps (Figure 4B–F,H–L) correspond to the models 1, 2, and 3, estimated from NMS-PK analysis, respectively.

**Figure 5:**
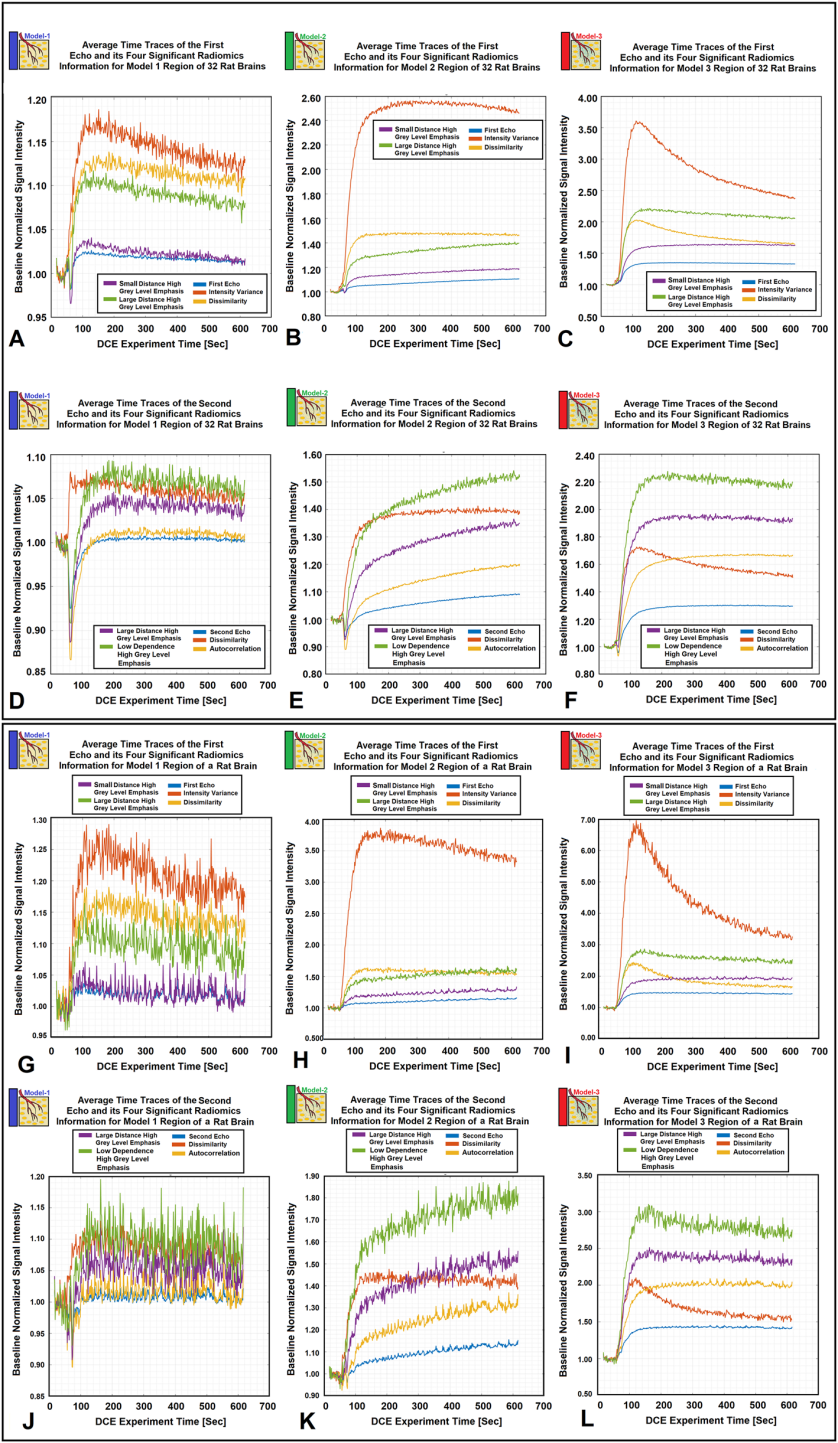
Subfigures (**A**–**C**) demonstrate the baseline normalized time traces of different information modalities (1st echo and its four corresponding significant radiomics information) averaged over three different model choice regions for all 32 animals. Subfigures 5D-F demonstrate the baseline normalized time traces of different information modalities (2nd echo and its four corresponding significant radiomics information) averaged over three different model choice regions for all 32 animals. Subfigures 5G-I demonstrate the baseline normalized time traces of different information modalities (1st echo and its four corresponding significant radiomics information) averaged over three different model choice regions for an individual rat brain. Subfigures (**J**–**L**) demonstrate the baseline normalized time traces of different information modalities (2nd echo and its four corresponding significant radiomics information) averaged over three different model choice regions for an individual rat brain.

**Figure 6:**
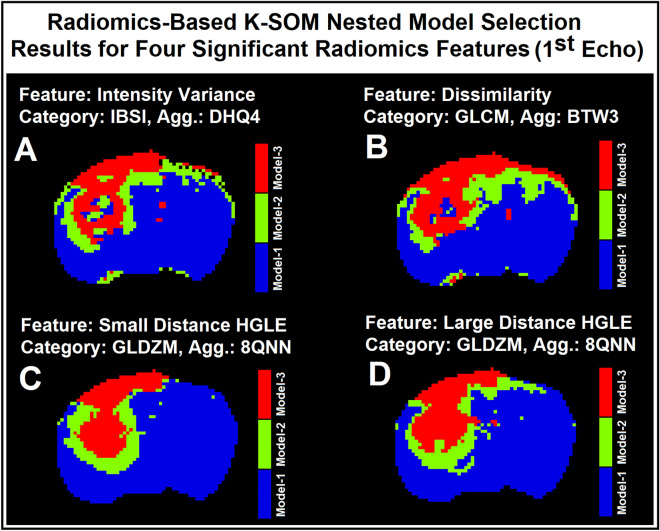
Subfigures (**A**–**D**) illustrate four examples for the model choice maps estimated from the significant radiomics information of the 1st echo. Each map was estimated by its corresponding radiomics-based K-SOM NMS analysis at probability threshold of 0.50.

Figure 6A–D illustrate four model choice maps estimated by the K-SOM of the significant radiomics information of the 1st echo. Each model map was estimated by its corresponding radiomics-based K-SOM NMS analysis at a probability threshold of 0.5. The time trace information (raw DCE-MRI and their respective four significant radiomics features) of each voxel was fed into its corresponding trained and model-labeled K-SOM and its BMU winning node was estimated and identified on the feature space. Then, the winning node was projected on the fused probability map of its respective information modality and the three chances/probabilities (corresponds to three NMS) for that voxel were calculated. Figure 5A–C demonstrate the baseline normalized time traces of different information modalities (1st echo and its four corresponding significant radiomics information) averaged over three different model choice regions for all 32 animals. Figure 5D–F demonstrate the baseline normalized time traces of different information modalities (2nd echo and its four corresponding significant radiomics information) averaged over three different model choice regions for all 32 animals. Figure 5G–L demonstrate the temporal changes of the 1st and 2nd echoes along with their respective significant radiomics features for the three models measured from an individual rat brain. Figure 6A–D illustrate four model choice maps estimated by the K-SOM of the significant radiomics information of the 1st echo. Each model map was estimated by its corresponding radiomics-based K-SOM NMS analysis at probability threshold of 0.5.

Table 4A and B demonstrate the average values of AUC, Balanced Accuracy, and F1 Score along with their respective confidence intervals estimated for the outer loop (unseen test cohorts) of the k-fold NCV (k = 10) for the two raw DCE-MR information and their 13 respective radiomics features. The bold features in this table refer to the discriminant radiomics features for the two echoes identified by the SCA.

**Table 4:** Table A illustrates the mean values of AUC, Balanced Accuracy, and F1 Score along with their confidence intervals estimated for the outer loop (unseen test cohorts) of the k-fold NCV (k = 10) for the first echo and its 13 radiomics features. Table 4B illustrates the same information for the second echo and its respective 13 radiomics features.

Feature category	Feature name	Feature aggregation technique	AUC	AUC lower confidence bound	AUC upper confidence bound	Balanced accuracy	Balanced accuracy lower CB	Balanced accuracy upper CB	F1 score	F1-score lower CB	F1-score upper CB
(A)											
Mean intensity	IBS	DHQ4	0.7488	0.7300	0.7675	0.6301	0.6172	0.6430	0.6717	0.6566	0.6867
** Intensity variance**	**IBS**	**DHQ4**	**0.7820**	**0.7662**	**0.7979**	**0.6921**	**0.6783**	**0.7059**	**0.7325**	**0.7162**	**0.7489**
Intensity skewness	IBS	DHQ4	0.6832	0.6657	0.7008	0.5975	0.5806	0.6145	0.6325	0.6112	0.6538
Intensity kurtosis	IBS	DHQ4	0.6671	0.6411	0.6930	0.5877	0.5664	0.6089	0.6195	0.5924	0.6466
** Dissimilarity**	**GLCM**	**BTW3**	**0.8242**	**0.8104**	**0.8380**	**0.7186**	**0.7078**	**0.7294**	**0.7583**	**0.7456**	**0.7711**
Autocorrelation	GLCM	BTW3	0.7631	0.7443	0.7820	0.6373	0.6252	0.6494	0.6801	0.6661	0.6941
Small distance low grey level emphasis	GLDZM	8QNN	0.6304	0.5824	0.6784	0.5726	0.5425	0.6026	0.5968	0.5556	0.6381
** Small distance high grey level emphasis**	**GLDZM**	**8QNN**	**0.7737**	**0.7561**	**0.7913**	**0.6572**	**0.6453**	**0.6692**	**0.6980**	**0.6838**	**0.7123**
** Large distance high grey level emphasis**	**GLDZM**	**8QNN**	**0.7813**	**0.7633**	**0.7993**	**0.6615**	**0.6497**	**0.6733**	**0.7037**	**0.6899**	**0.7176**
High grey level count emphasis	NGLDM	8QNN	0.7602	0.7451	0.7753	0.6375	0.6272	0.6479	0.6804	0.6683	0.6925
Low dependence low grey level emphasis	NGLDM	8QNN	0.5682	0.4885	0.6479	0.5321	0.5029	0.5614	0.5353	0.4903	0.5803
Low dependence high grey level emphasis	NGLDM	8QNN	0.7434	0.7292	0.7577	0.6281	0.6157	0.6405	0.6695	0.6550	0.6841
High dependence low grey level emphasis	NGLDM	8QNN	0.7424	0.6995	0.7854	0.6345	0.6110	0.6580	0.6757	0.6465	0.7048
** Raw DCE MR information**	**First echo**	**NA**	**0.7619**	**0.7436**	**0.7803**	**0.6412**	**0.6304**	**0.6520**	**0.6844**	**0.6717**	**0.6970**
(B)											
Mean intensity	IBS	DHQ4	0.7388	0.7233	0.7543	0.6230	0.6124	0.6336	0.6634	0.6508	0.6760
Intensity variance	IBS	DHQ4	0.7006	0.6795	0.7217	0.6138	0.5984	0.6292	0.6526	0.6337	0.6714
Intensity skewness	IBS	DHQ4	0.6713	0.6551	0.6874	0.5861	0.5695	0.6027	0.6181	0.5968	0.6394
Intensity kurtosis	IBS	DHQ4	0.6648	0.6522	0.6773	0.5764	0.5581	0.5946	0.6054	0.5816	0.6292
** Dissimilarity**	**GLCM**	**BTW3**	**0.8129**	**0.7930**	**0.8328**	**0.7204**	**0.7078**	**0.7331**	**0.7606**	**0.7454**	**0.7759**
** Autocorrelation**	**GLCM**	**BTW3**	**0.8008**	**0.7834**	**0.8182**	**0.6834**	**0.6710**	**0.6957**	**0.7242**	**0.7096**	**0.7389**
Small distance low grey level emphasis	GLDZM	8QNN	0.5445	0.4907	0.5982	0.5496	0.5248	0.5745	0.5678	0.5332	0.6024
Small distance high grey level emphasis	GLDZM	8QNN	0.7312	0.7114	0.7510	0.6149	0.6027	0.6272	0.6536	0.6387	0.6685
** Large distance high grey level emphasis**	**GLDZM**	**8QNN**	**0.8156**	**0.7995**	**0.8316**	**0.6987**	**0.6881**	**0.7094**	**0.7396**	**0.7269**	**0.7523**
High grey level count emphasis	NGLDM	8QNN	0.7420	0.7228	0.7611	0.6255	0.6112	0.6399	0.6661	0.6490	0.6831
Low dependence low grey level emphasis	NGLDM	8QNN	0.5875	0.5388	0.6362	0.5356	0.4872	0.5840	0.5392	0.4678	0.6105
** Low dependence high grey level emphasis**	**NGLDM**	**8QNN**	**0.8078**	**0.7944**	**0.8213**	**0.6869**	**0.6766**	**0.6973**	**0.7276**	**0.7151**	**0.7401**
High dependence low grey level emphasis	NGLDM	8QNN	0.6546	0.5980	0.7112	0.5998	0.5710	0.6285	0.6308	0.5933	0.6683
** Raw DCE MR information**	**Second echo**	**NA**	**0.7514**	**0.7365**	**0.7663**	**0.6324**	**0.6232**	**0.6416**	**0.6740**	**0.6632**	**0.6849**

The bolded features in both tables (A–B) refer to the discriminant features identified by the Silhouette clustering analysis.

## Discussion

The outcome information was tagged on the feature space according to the NMS theory to reveal the level of agreement and association between the detected structures and the outcome information. The K-SOM technique is an unsupervised learning method which enables detection and preservation of the topological relationship of the training dataset based on the data similarity. This implies that the underlying properties of the feature space are uncovered to discover associations that are not easily identified from the raw signal.

We investigated the superiority of the raw DCE-MRI information compared to their respective radiomics information (total of 28 information modalities: the two echoes and their respective radiomics features, 13 feature per echo) to explain different pharmacokinetic-based pathophysiological states of tissues according to the concept of nested model selection theory. In this study, no predictive model was developed, while for each echo, we compared the pathophysiological-based clustering power of raw DCE-MRI information against 13 respective radiomics features using an unsupervised (no forced/supervised learning) similarity analysis. The goal was to evaluate, reveal, and compare the pathophysiological information content (such as distribution of the information and their uncertainty levels or penumbra effects) of different information modalities on the feature space.

We investigated the information content of different DCE-based radiomics features extracted from 32 treatment-naïve rat brains using convolutional radiomics information analyzed by an unsupervised K-SOM algorithm^106,107,121,122^. The aim of the study was to reveal and explore the physiological associations of these features with different characteristics of underlying pathologies (tumor and normal tissue) compared to their respective raw DCE-MRI information.

The time traces of radiomics information were projected on a two-dimensional topology space to reveal any potential non-linearities, similarities, and dissimilarities of the spatiotemporal structures of the data in the form of clusters in the K-SOM feature space. The notable characteristic of K-SOM technique is that the detailed structures of input vectors that are close, and similar in high dimensional space are mapped to nearby nodes in the topology space of the network. It is in essence, an unsupervised method for data dimensionality reduction and grouping the information, as it maps high-dimension inputs to a low dimensional discretized representation while conserving the underlying structures of its input space^106,107,121,122^.

The K-SOM analysis technique used in this study, not only preserved and mapped the characteristics of each information modality (time traces of radiomics features and raw DCE-MRI information) at different time points to a feature space, but also correspondingly compared the information of different time points of these modalities during the mapping process using a competitive learning approach. In this study, the recruitment of such an unsupervised mapping revealed different degrees of associations^106,107^ among dynamic information modalities extracted from tumor and its soft surrounding normal tissues with their respective pathophysiological states. Figure 4B–F and H–L clearly demonstrate how different time traces of information modalities (two raw-DCE MRI and their four respective significant radiomics features) corresponding to different pathophysiological states are grouped together based on their structural similarities at different timepoints. This generated distinct clusters with less scattered fragments of information without supervision on the K-SOM’s feature space. The blue, green, and red zones on the fused K-SOM’s topology maps (Figure 4B–F and H–L) correspond to the models 1, 2, and 3, estimated from NMS-PK analysis, respectively.

We recruited the NMS analysis along with a series of statistical analyses^113–115^ and unsupervised SCA^111,112^ which were repeated at different levels of specificities or probability thresholds to measure the stability of the distinction power (among different nested models) of each information modality on the K-SOM’s feature space. The NMS and PK analysis results of the standard Patlak analysis were used as the source of truth for labeling the K-SOM topology maps and evaluation of the SCA.

Figure 3A and B, clearly demonstrate the sensitivity and stability of the distinction power of different information modalities (1st and 2nd echoes of raw DCE-MRI information and their 14 respective radiomics features). This allowed us to evaluate and measure the cohesiveness, integrity, and separation of the three clusters (labeled from conventional PK analysis for the three nested models, Section “Nested model selection and observation equations for pharmacokinetic analysis”) at different specificities or probability thresholds (from 0.01 to 1.00, corresponding to 100 probability intervals) based on the NMS concept.

The statistical analysis results showed that there are eight significant radiomics features (four features for each echo) that outperform the raw-DCE-MR information in discrimination of the three nested model regions (labeled from conventional PK analysis for the three nested models, Section “Nested model selection and observation equations for pharmacokinetic analysis”). These significant radiomics features provide a higher certainty in discrimination of the three physiologically nested model regions within tumors and their soft surround normal tissues at different specificity levels (100 different probability thresholds based on NMS analysis). This resulted in a more precise physiological characterization of normal tissue and tumor microenvironment and heterogeneity.

The time traces of significant radiomics information shown in Figure 5A–F strongly support the physiological associations of the information contents of the different information modalities (two raw-DCE MR information and their respective radiomics features) for characterization of the pathophysiological state of the regions associated with the three different models^64,77,92,123,124^, after CA administration. In these subfigures, the differences among the rise time and slope of the time traces of the radiomics information compared to their respective raw-DCE-MR information (1st or 2nd echo), for different model choice regions, strongly confirms the value and distinction power of the identified radiomics features for characterization of tumor heterogeneity and microvascular characteristics. Interestingly, the time traces of the *Intensity-Variance* and *Dissimilarity* features demonstrate clear evidence of the CA washout trend from extravascular extracellular space after ~ 180 s of the experiment. This strongly supports the expected PK trend associated with the Model 3 regions formulated in the washout kernel of observation Eq. (12). Similarly, for the 2nd echo, the time traces of *Low Dependence High Grey Level Emphasis* and *Dissimilarity* features show similar trends that support the expected PK trend for the CA washout for Model 3 regions.

Figure 6A–D illustrate four model choice maps estimated from the significant radiomics information of the 1st echo. Each map was estimated by its corresponding radiomics-based K-SOM NMS analysis at probability threshold of 0.5. As shown in these subfigures (Figure 6A–D versus 2L or 2F), the radiomics-based K-SOMs produced stable maps of nested model regions with Model-1 regions less impacted by the dispersion effects due to arterial input function and magnetic field gradient effect. Thus, less mis-classification for Model-1 and 2 regions.

Since the physiological properties of brain tissue are mostly constant across species and pathologies^125–127^, the associations of radiomics information with brain tissue physiology revealed in this study, can be expected to reliably scale and translate to human Glioblastoma (GBM). Physiological and mechanical properties of tumor have been shown to characterize heterogeneity in gene expression, and phenotype for Glioblastoma (GBM)^52,54,63,70,128–133^. Many studies^30–36,39–41,134–139^ have developed different DCE-based radiomics model for characterization of tumor’s phenotype, vascular properties, and regional heterogeneity in the field of cancer research. However, none of these studies have explored the power of PK-based spatiotemporal radiomics information for characterization of tumor’s pathophysiological condition according to a DCE physiological-based model as well as its comparison with raw DCE MR information that includes the full spectrum of information content. The results of this pilot study can help generate novel MR-based radiomics estimators to interpret and translate estimation of the physiological properties of solid tumors and soft surrounding normal tissues in embedded tumors—initially focused on GBM, but extendable to other tumors.

Recent studies^40,139^ have recently shown the association between the subregional PK-based radiomics information of breast tumor and its histological characteristics. These studies also shown that compared to the entire tumor region, subregional PK-based radiomics information can enhance the predictive performance of the radiomic models. Many studies^30–41,134–137,139,140^ have developed different DCE-based radiomics model for characterization of tumor’s phenotype, vascular properties, and regional heterogeneity in the field of cancer research. These studies mainly focused on the radiomics analysis of the parametric maps (such as K^trans^, v_p_, etc.) and their associations with different outcome of interests. For instance, Fusco et al.^32^ have discriminated between benign and malignant breast lesions using several classifiers and radiomics features extracted from DCE-MRI images. In a similar study, Zhao et al.^141^ constructed different radiomics models as well as an ensemble model from DCE-MRI and Mammography images to improve diagnosis of breast cancer. In another study^142^, Peng et al. explored the association of pretreatment DCE-MRI radiomics signatures with pathologic complete response (pCR) to neoadjuvant therapy (NAT) in breast cancer. However, none of these studies have explored PK-based spatiotemporal radiomics information for characterization of tumor pathophysiology using a DCE-based model and comparison against raw DCE-MR information. This is the first such study.

This highlights the importance of the inclusion of local information in characterization of tumor heterogeneity and strongly supports the dynamic convolutional radiomics analysis used in this study.

In general, the value of DCE-MRI experiment in tissue characterization mainly relies on revealing the contrast enhanced (CE)-based information content of underlying pathology. The normalized CE ratio threshold (CER > 1.2)^105^ used in this study, excluded a series of radiomics feature maps with non-significant contrast-based information content which may still have adequate signal-to-noise ratio (SNR) for radiomics analysis. Such a feature exclusion facilitated the feature engineering part of the study and drastically reduced the computation time and complexity of the statistical analysis. To this end, decreasing the CER threshold^105^ would increase the number of radiomics feature maps with adequate SNR for feature engineering that might not have enough DCE-based or CE-based information. Therefore, finding an optimal CER value for exclusion or inclusion of the radiomics features that are less associated with the DCE-MR information warrants further investigation.

Dual Gradient Echo (DGE) pulse sequence used in this study, allows computation of *T*_*1*_ and *T*_2_* as approximately pure components. This is essential for estimation of CA concentration (*ΔR*_*1*_ and *ΔR*_2_*).

The ability to estimate pure components is an advantage to the Spoiled Gradient Echo (SPGRE)-based DCE-T_1_ imaging. Specifically, where nonlinearities in the MR measurement of ΔR_1_ due to limited access of water in tissue compartments distal to the CA and the *T*_2_* contrast competing with *T*_*1*_ contrast. These effects can substantially bias estimates of tissue CA concentration. Another advantage of the DGE sequence is the relatively short time interval between image acquisitions (~ 1.55 s in this study).

The T_2_* relaxation effect is associated with the decay or dephasing of the transverse magnetization caused by a static and local magnetic field inhomogeneities. A longer echo time (T_E_) would allow more dephasing of the signal. Thus, T_2_* dephasing directly affects the uncertainty level and heterogeneity content of the raw DCE-MRI signal due to the local and static magnetic field inhomogeneity. The echo time (T_E_ = 4 ms) for the 2nd echo images was twice as long as the 1st echo images. Thus, compared to the 1st echo images, the 2nd echo images exhibit more signal heterogeneity with less signal to noise ratio (SNR). As shown in Table 2, the *Dissimilarity* feature (DSC% = 42%, p < 0.001), extracted from the 2nd echo showed less association (%7 lower DSC%) with the model choice information compared to the same feature (Dissimilarly, DSC% = 49%, p < 0.001) extracted from the 1st echo. This confirms our expectation from the higher dephasing effect associated with the 2nd echo images.

The DSC% of the Large Distance High Grey Level Emphasis feature (from GLDZM feature category) extracted from 2nd echo being almost three times higher than the same feature extracted from 1st echo, implies that compared to the 1st echo, the 2nd echo might contain higher or richer information for tissue characterization. This might be due to fact that the 2nd echo may contain some information, relative to the 1st echo, about the size and shape of the spaces (such as cell tortuosity information) that CA is occupying. Thus, despite the 2nd echo having less Signal-to-Noise ratio compared to the 1st echo, its information content seems much higher.

In this study, the convolutional radiomics analysis was not performed with different convolutional window sizes and we only performed the analysis using a 5 × 5 convolutional window size. We didn’t increase the size of the convolutional/sliding window to avoid high computation time and to prevent high spatial aggregation of the information inside the sliding window which ‘smears’ the data around the central voxels and would lead to loss of spatial details or high-frequency information. Of note, this is a spatiotemporal radiomics analysis, and the values of the elements in the textural matrices (feature space) highly depend on the saturation level of the tumor (contrast-enhancement ratio, as discussed in Section “Feature engineering, kohonen self-organizing map, and clustering analysis”) during the DCE-MRI experiment. As for the typical values of the elements of the textural matrices, in general, most of the elements of the symmetrical GLCM matrices are ranged between 0 to 4 or maximums of 6 to 8.

The range of the elements falls in the order of the contrast enhancement ratio (~ 1.2 or 20% that corresponds to about 5 times of enhancement) and would allow the effective capture of the local spatial variations of the intensity. It is also wide enough to capture the information relevant to the “intensity-change” that is caused by the extravasation of the contrast agent concentration (CA) during the DCE-MRI experiment with the same order as mentioned in Section “Feature engineering, kohonen self-organizing map, and clustering analysis”. As the intensity of the sliding window increases temporally, and it becomes saturated (CA concentration increases inside the tumor zone), the non-zero elements of the feature space (textural matrix) would not only show changes in their cluster shape around its diagonal elements, but also are shifted toward the lower section of the matrix. In fact, the more saturation effect inside the sliding window will result in less variations in the information occurrence inside of its textural matrix. Furthermore, choosing a relatively large quantization level (gray-level, 128) would enable a wide range of intensity change (due to the tumor leakage) throughout the DCE-MRI experiment and help with the revelation of the DCE informative features. As mentioned in Section “Feature engineering, kohonen self-organizing map, and clustering analysis”, the contrast enhancement ratio (CER) plays a central role in the DCE-MRI experiment, and we used the normalized CER averaged over 32 studies to exclude the radiomics information modalities with low normalized contrast enhancement ratio (NCER < 1.2) that were not DCE informative. Recruitment of different convolutional window sizes can capture and extract different levels of information details from raw DCE-MRI information. This may produce time trace of radiomics information with weaker or stronger associations to the local variations of the tissue regarding its contrast enhancement, spatial heterogeneity, and the physiologically inspired effects, such as washout of CA from extra-cellular extra-vascular volume, etc. We performed all the convolutional radiomics analyses using a symmetrical convolutional window (w_x_ × w_y_) with size of 5 × 5. As the size of the convolutional window increases, the less local information is captured and incorporated into the radiomics feature information and the aggregation of the information around each central voxel would increase. This would result in the loss of information heterogeneity that is captured and extracted by the radiomics analysis. The effect and trade-off for choosing different sizes of the convolutional window and the optimal Chebyshev distance which is relevant to the sampling frequency of the information on the analysis results requires further investigation.

As to translation to the clinic, the goal of this study was not to develop any translatable model. The scope of this study was to reveal the power and ranking of different DCE-MRI based components (1st and 2nd echoes which are associated with T_1_ and T_2_* based information, respectively) to explain the pathophysiological and microvascular properties of normal and cancerous tissues.

The MRI features of the U-251N animal model tumors correlate with human GBM, including a necrotic center, poorly demarcated, infiltrative tumor borders, and an enhanced rim on T2-weighted imaging (one of the main components of the Dual Gradient Echo used in this study); on post-contrast T1-weighted images an intense rim is observed similar to human GBM^126^. It has already been shown that in murine models, DCE-MRI measures of vascular permeability^59,60,63,64,70,77,82,94,96–99,123,129,143–148^ agree with those of human studies^77^.

Our group is one of the few studying animal tumors using the dual gradient-echo (DGE) pulse sequence. In the construction of vascular permeability and parametric maps, such a sequence allows the separation of T_1_ and T_2_* effects. In an information-centered approach, the data contains additional information concerning the extravascular regime of the tumor which can be explained and modeled by these two components. Thus, given the fact that cerebral physiology and mechanical properties are relatively constant across species^125–127^, the analysis results of this study can shed light on different DCE-MR based components and their values in characterization of microenvironmental information of tumors. Such information has an excellent potential for positively informing the assessment and treatment of deadly tumors in humans and should be considered as vital information to the clinical research community for understanding the underlying physiology of solid tumors. Thus, this work, if repeated and fine-tuned on the clinical pules sequence (such as spoiled gradient-recalled echo, SPGRE) with different time resolution, is very likely to be translatable to clinical studies.

In animal models, DCE-MRI, Pharmacokinetic analysis shows promise as a stable descriptor of tumor physiology^59,70^, and of physiological reaction to treatments^61,71–76^. It also shows promise for describing stresses, flows, and fluxes, but it is newer and needs validation. In the clinical setting, DCE-MRI has also been established as a useful, noninvasive, and non-ionizing method for quantitative evaluation and characterization of tumor microvasculature and permeability parameters^59–69^. This pilot study supports the promise and value of the radiomics information as a descriptor of tumor physiology with a higher certainty in discriminating the three physiologically nested-model regions in and around tumors. This would result in a more precise physiological-based characterization of tumor heterogeneity and normal tissue. In recent years, many studies have investigated the development of various deep learning and Convolutional Neural Networks (CNNs) to generate more accurate and stable estimates of PK vascular parameters by extracting time-dependent features from DCE-MRI^62,149–154^. Compared to the recent studies^62,149–154^, one of the novel components of this study is the incorporation of the nested model selection concept into the radiomics analysis. The NMS is considered as the first key step in predicting the PK parameters^64,77^ and in general, for DCE-MRI data analysis, one of the main challenges is choosing the best PK model among competing models to describe the behavior of the time trace of CA concentration in DCE MR experiments.

The goal of this study was not to develop any predictive models while the focus was to investigate the value of the radiomics information for association of the image information to the pathophysiological information of underlying tissue.

This study confirmed the superiority of the radiomics-based K-SOMs compared to conventional PK analysis of raw DCE-MRI information in connecting different pathophysiological states (model regions) with imaging information. Given the results of this study, our group is currently working on the development of a supervised predictive model that incorporates unsupervised radiomics-based K-SOM (constructed from different radiomics features at different NMS probability thresholds) for a more accurate prediction of the NM regions in PK analysis.

We used a competitive learning algorithm^108–110^ along with BMU strategy to identify the winner nodes for a 10X10 hexagonal topology^106–110,121^. However, recruitment of different topology sizes can affect the quality of the K-SOM algorithm in detecting the association and distinction of the NM clusters on the feature space.

As for the SC analysis, we did not make any assumption regarding the distributions of the three model-based clusters and their changes at different probability thresholds on the feature space. The K-SOM hit map and its respective clustering analysis heavily relies on the cumulation of number of hits and grouping of the data on the feature space based on their similarities. To reveal potential linear/non-linear trends and structures of all the clusters’ distributions and their changes at different probabilities corresponds to different sensitivities/specificities, an SC analysis (see Table 3) with variable thresholds was performed. This allows the discovery of any unexplored insights of the cluster information and their distinction power (the agreement between the revealed structures and the labels from the NMS) on the feature space. Thus, like the ROC analysis, the SC analysis with variable threshold allowed us to perform an empirical sensitivity analysis to calculate the distinction power of the three major clusters formed at different probability levels calculated from the K-SOM hit maps.

The results of this study can be used for further modeling (see Fig. 5) to improve the PK analysis of DCE-MRI data of rat brain. As a continuation of this study, our group is currently working on the predictive analytics and feature imitating network (FIN) technique^155^ to refine the features at the feature engineering stage and to construct more robust adaptive models to refine the estimation of the PK parameters, as well as predicting microvasculature properties of rat brain tissues. The goal is to combine the transfer learning technique with the FIN to generate imitated feature maps from the significant features revealed in this study to improve the predictive power of the adaptive models. As future work, we believe that if the K-SOM is combined with the Transfer Learning with Feature Extraction Modules Network (TrFEMNet)^156^, Feature representations from General Feature Extraction Module (GFEM)^156^, and Specific Feature Extraction Module (SFEM)^156^, it would generate more stable and in-depth results for further modeling. The major role for the transfer learning will come into play when the significant radiomics features/biomarkers discovered by this study (in form of K-SOM topology or feature space) are used as a learned representation for physiological knowledge transfer into the clinical setting that uses the same imaging concept with a slightly different DCE-MRI pulse sequence (SPGRE). This will preserve the properties or the potential pathophysiological structures of the DCE data that are similar across rat and human brains for more robust modeling. Significant radiomic features will be identified that will be independent from both pharmacokinetic and mechanical parameterizations, but correlated with the local tissue properties (e.g. cell density, and extracellular matrix) identified by histopathology or nested model selection theory.

The k-fold NCV analysis results shown in Table 4 are strongly in agreement with the results obtained by the Silhouette clustering analysis (SCA). As shown in this table, the discriminant features (bolded features in Table 4A and B as follows: Dissimilarity, Intensity Variance, Large Distance High Grey Level Emphasis, and Small Distance High Grey Level Emphasis features for the first echo, and Dissimilarity, Large Distance High Grey Level Emphasis, Low Dependence High Grey Level Emphasis, and Autocorrelation features for the second echo) identified by the SCA, show higher performances (see their AUCs, Balanced Accuracies, and F1 Scores along with their confidence intervals) for classification of the three nested models compared to other radiomics features and their respective raw-DCE-MRI information (1st and 2nd echoes).

Of note, the k-fold NCV analysis performed in this study, reveals the information content of different information modalities and their stabilities to describe the three pathophysiological states of the tissue. As shown in Table 4, the average values of the evaluation metrices for all the information modalities reported for the outer loops of the k-fold NCV (see Table 4A,B) are less than 0.80. This is due to the fact that the unsupervised data analysis recruited in this study, only reveals the association of different information modalities with the three nested models. Thus, the K-SOMs and their corresponding feature spaces developed by the training cohorts (in the inner loop) can reveal signal similarities and dissimilarities which are highly susceptible to different effects (such as noise, arterial input function dispersion or delay^157,158^ in normal tissue, contrast agent arrival time differences among different temporal signals of different information modalities, etc.). These systematic and random effects can also result in uncertainties in the classification models (source of truth). In contrast to the unsupervised data analysis, in the supervised model development, a set of optimal classification thresholds can be chosen according to the desired level of the misclassification errors and the error tolerance among different classes/models. Thus, for supervised models it is expected to see less overlap among different classes/models which leads to higher classification performance. The goal of this study was to connect and compare the raw-DCE-MRI information and their respective radiomics features extracted from tumor and its surrounding normal tissues with the conventional NMS analysis results. Of note, this allows the incorporation of many effects, i.e., systematic, and random-like errors, both in the target and input signals of the K-SOMs networks. Regardless of the type of the adaptive model (supervised/unsupervised) used for this study, these effects would not usually be included in model simulations, are not easily evaluated in those processes, and thus might introduce bias in evaluating the performance of an adaptive model when applied to practical situations.

Indeed, K-SOM algorithm is considered as one of the first self-organizing algorithms^121^ that groups multi-dimensional data in the form of clusters on the feature space with no supervision for the purpose of visualization, clustering analysis, and dimensionality reduction^106–110,121,159^. There have been several improvements^121,159^ made to the original algorithm proposed by Kohonen, such as the Batch-SOM^121^, Dot-Product-SOM^121^, a SOM which identifies a linear mixture of model vectors instead of winner nodes^122^, and a O(log_2_M)-SOM^160^, that works based on a stratification technique which inherently deals with information propagation to neighborhood nodes on the feature space. These approaches mainly improve the optimization and feature information representation on the feature space. There are other algorithms that can also help improve the original K-SOM’s performance, convergence, stability, and its adaptability on different problems. These include the Growing Grid SOM^161^, Growing Neural Gas^162^ algorithms, and Hierarchical-SOM^163^, which incorporate an additional layer of nodes to automatically determine of the optimal topology size of the K-SOM based on the properties of the original algorithm. While these altered algorithms demonstrate some clear advantages over the original K-SOM such as improved data representation, memory and speed optimizations, etc., they might introduce some bias to the generated clusters as well as the quality of the feature visualization on the feature space. Further investigation of the effects of different K-SOM architecture sizes and algorithms and their impact on the K-SOM efficiency on the radiomics data clustering analysis is warranted.

## Conclusions

This study incorporates a spatiotemporal analysis of the radiomics information of cerebral tumor and surrounding normal tissue using DCE-MRI studies. The methods have enabled evaluation of the time-varying information^164^, content of the raw data and subsequent associations with physiological parameters on a voxel-by-voxel basis. K-SOM Radiomics-based models constructed in this study can be used to identify *missing information not estimated by conventional DCE-MRI PK analysis.* This study is the first to discover the potential value of radiomics information to explain physiological parameters of embedded and solid tumors in an animal, brain tumor model. We have used standard model selection techniques and IBSI-approved radiomics analyses to circumvent the reproducibility and biasing effects of compartmental modeling in the pharmacokinetic analysis. This study provides detailed information to describe the physiological properties of solid tumors and surrounding normal tissues. If it is fine-tuned and translated for human use, such tools can significantly improve the clinical diagnostic and prognostic decisions made for patients with high grade gliomas. This research has potential for positively informing the assessment and treatment of deadly tumors in humans.

This study is the first to develop radiomics-based predictors of physiological parameters of GBM tumors in an animal model that recruits standard model selection techniques^64,77^ and IBSI approved radiomics analysis to circumvent the reproducibility and biasing effects of radiomics analysis and compartmental modeling in PK analysis. This study supports the promise and value of the radiomics information as a descriptor of tumor physiology with a higher certainty in discrimination of the three physiologically nested-model regions in and around tumors, resulting in a more precise physiological-based characterization of tumor heterogeneity and normal tissue. This is an important step toward spatiotemporal radiomics-based characterization of brain regions which is fundamental in staging of aggressive tumors, evaluation of their response to different treatments, design and optimization of DCE-MR imaging, and PK modeling to improve tumor characterization.

## Data Availability

All imaging data used in this investigation along with programming codes and results can be shared upon request to the corresponding and senior authors, Dr. Hassan Bagher-Ebadian and Dr. Indrin J. Chetty, respectively. Of note, the animal data used in this study, is collected as a part of the NIH grant (R01, NCI/NIH R01-CA218596) and can be shared upon reasonable request sent to the principal investigators of the grant, Drs. James R. Ewing and Stephen Brown (co-authors). Also, a MATLAB version of the ROdiomiX software which is a validated software for radiomics analysis of medical images in radiation oncology (developed and validated according to the IBSI guidelines by Drs. Bagher-Ebadian and Chetty in the year 2021^165^) has already been publicly shared at the GitHub website: (https://github.com/Ebadian-HFHS/ROdiomiX).
